# Broken Heart Syndrome in a Patient on Maintenance Hemodialysis

**DOI:** 10.1177/2324709617713512

**Published:** 2017-06-12

**Authors:** Sukhdeep Bhogal, Vatsal Ladia, Puja Sitwala, Kailash Bajaj, Vijay Ramu, Timir Paul

**Affiliations:** 1 East Tennessee State University, Johnson City, TN, USA

**Keywords:** broken heart syndrome, Takotsubo cardiomyopathy, end-stage renal disease, hemodialysis

## Abstract

**Context:**Broken heart syndrome or Takotsubo cardiomyopathy (TC) is a disorder characterized by transient left ventricular apical ballooning that almost invariably precedes emotional or physical stress. Although the patients with chronic kidney disease on hemodialysis have shown to exhibit sustained activity of sympathetic nervous system, the presentation of TC in these patients is a rare entity with few case reports in the literature. **Case Report:** A 75-year-old female with past medical history of end-stage renal disease presented with chest pressure and heaviness that started during her maintenance hemodialysis session. Electrocardiogram showed ST elevation and T wave inversion in V3-V6 leads. Emergent left heart catheterization was done that showed normal coronaries and akinesis of apical left ventricle wall consistent with TC. She was started on maximal medical management and underwent hemodialysis the next day without recurrence of the symptoms. **Conclusion:** TC may an underdiagnosed entity in patients on hemodialysis. However, it should be considered in the differential diagnosis in hemodialysis patients, particularly who presents with chest pain and/or symptoms.

## Introduction

Broken heart syndrome or Takotsubo cardiomyopathy (TC) is a disorder mimicking acute coronary syndrome characterized by transient regional wall motion abnormality in the absence of coronary artery disease. The entity is often preceded by emotional or physical stress. Patients with chronic kidney disease and/or on hemodialysis have been found to be associated with increased risk of cardiovascular mortality.^1^ However, TC in the setting of maintenance hemodialysis is a rare entity with few case reports in the literature.

## Case Report

**Figure 1.**
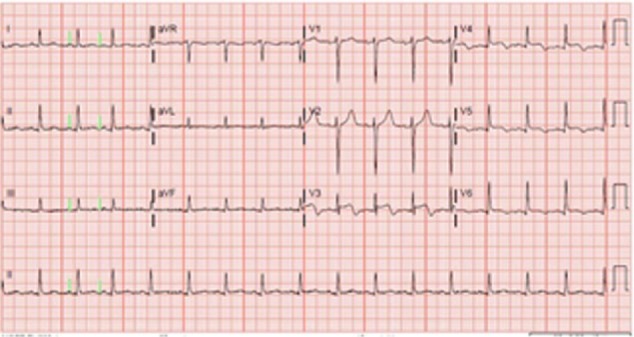
Electrocardiogram showing ST elevation and T wave inversions in V3-V6 leads.

**Figure 2.**
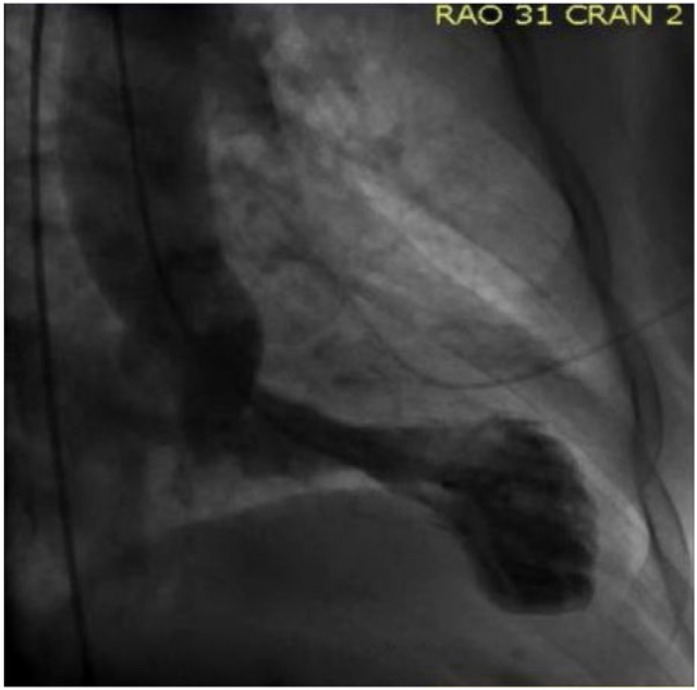
Left ventriculogram demonstrating akinesis of apical left ventricle wall consistent with Takotsubo cardiomyopathy.

A 75-year-old female presented to our hospital with chest pressure and heaviness that started during her maintenance hemodialysis session. She had past medical history of end-stage renal disease (ESRD) on hemodialysis for the last 5 years, diabetes, and hypertension. She reported recent hospitalization of her husband for an acute illness. Physical examination was normal except for lower extremity edema. On presentation, her blood pressure was 122/61 mm Hg, heart rate of 71 beats/minute, and respiratory rate of 18/minute. Initial laboratory tests were remarkable for hemoglobin of 9.1 g/dL and creatinine of 2.21 mg/dL. Electrocardiogram showed ST elevation and T wave inversion in V3-V6 leads (Figure 1). Troponin levels were also found to be elevated 8.06 ng/mL (reference range = 0.00-0.02 ng/mL). Emergent left heart catheterization (LHC) was done that showed normal coronaries but the ventriculogram revealed mildly reduced left ventricular (LV) systolic function with ejection fraction of 45% to 50% and akinesis of apical LV wall consistent with TC (Figure 2). Echocardiography demonstrated akinetic apex of LV with ejection fraction of 65%. She underwent hemodialysis the next day after undergoing LHC without recurrence of symptoms. She was discharged on atorvastatin, metoprolol, and lisinopril and follow-up course was uneventful. The follow-up echocardiogram was performed at 2 months, which was consistent with the resolution of the akinetic LV apex with the ejection fraction of 68%.

## Discussion

TC or broken heart syndrome is a disorder characterized by transient left ventricular apical ballooning which mimics presentations of acute coronary syndrome but without any angiographic evidence of coronary artery disease.^2^ It is generally seen in postmenopausal women following an emotional or physical stress and/or critical illness.^3^ The prevalence is estimated to be approximately 1% to 2% of cases of acute coronary syndrome.^4^ The exact pathophysiology remains undetermined, but it has been postulated that inappropriate catecholamine release in relation to emotional stress may be the underlying pathological factor.^5^ The hypothesis is further supported by Giavarini et al in a retrospective study who reported that up to 11% cases of pheochromocytomas and paragangliomas may present as Takotsubo-like cardiomyopathy.^6^ Additionally, Wittstein et al demonstrated that plasma catecholamine levels were significantly high in patients with TC than patients with acute myocardial infarction.^7^ Moreover, the drugs with excessive catecholamine and beta receptor agonist effect may precipitate TC.^8^ Similarly, sustained activity of sympathetic nervous system and excess catecholamine release has been exhibited in patients with ESRD on hemodialysis^9,10^ in context to the development of TC in these patients. ESRD patients, in addition to physiological changes, also suffer from a significant psychological illness that can adversely affect their lifestyle.^11,12^ González-De-Jesús et al demonstrated that up to 55% to 60% of these patients exhibits either depressive or anxious symptoms based on the hospital anxiety and depression scale.^13^ Initiation of dialysis has also been reported as a triggering factor for TC in 4 patients.^14^ We believe activation of sympathetic nervous system releasing catecholamine, particularly in patients who have emotional stress, may be the potential cause of TC. So far, to the best of our knowledge, only 8 cases of TC were found after extensive literature search.^15-19^ Table 1 summarizes the data of TC in patients on hemodialysis. These patients have the age range from 54 to 84 years. Including our case, 7/9 (77.7%) patients are women. Interestingly, more than half of these patients (5/9; 55.5%) did not have chest pain. Considering this, it is worth to mention here that condition may be underdiagnosed considering its atypical or asymptomatic presentation. Nevertheless, all of these patients have an uneventful recovery.

**Table 1.** Summary of Data of Takotsubo Cardiomyopathy in Patients on Hemodialysis.

Authors	Age and Gender of Patient	Interval of Development of Symptoms	Past Medical History	Precipitating Factors	Duration of Hemodialysis	Outcome
Fukui et al^15^	84 years, female	Chest discomfort, several days before admission	Chronic nephritic syndrome and renal parenchymal hypertension	Smoking cessation	2 years	No recurrence of symptoms
Muratsu et al^16^	63 years, female	No symptoms, second day of hospitalization	Glomerulonephritis	Seizure	32 years	No recurrence of symptoms
Muratsu et al^16^	59 years, female	Fatigue on admission	Glomerulonephritis	Family illness	12 years	No recurrence of symptoms
Kusaba et al^17^	65 years, male	Severe left shoulder pain, sixth day of admission	Diabetic nephropathy	Meningitis and cervical epidural abscess	9 years	No recurrence of symptoms
Takemoto et al^18^	61 years, female	Severe chest pain during hemodialysis session	Glomerulonephritis	Cervical spondylosis surgery	20 years	No recurrence of symptoms
Shin et al^19^	Age range 54-68 years, 2 females and 1 male	Dyspnea on admission	Diabetes and hypertension	Pneumonia and hypoxia in 2 patients and colitis in third	—	No recurrence of symptoms
Current case	75 years, female	Chest pressure during hemodialysis	Diabetes and hypertension	Family illness	5 years	No recurrence of symptoms

## Conclusion

Takotsubo cardiomyopathy may be an underdiagnosed entity in patients on hemodialysis. However, it should be considered in the differential diagnosis in hemodialysis patients, particularly who present with chest pain and/or symptoms.

## References

[1] Sarnak MJ Levey AS Schoolwerth AC Kidney disease as a risk factor for development of cardiovascular disease: a statement from the American Heart Association Councils on Kidney in Cardiovascular Disease, High Blood Pressure Research, Clinical Cardiology, and Epidemiology and Prevention. Circulation. 2003;108:2154-2169. 14581387 10.1161/01.CIR.0000095676.90936.80

[2] Virani SS Khan AN Mendoza CE Ferreira AC de Marchena E Takotsubo cardiomyopathy, or broken-heart syndrome. Tex Heart Inst J. 2007;34:76-79. 17420797 PMC1847940

[3] Ueyama T Emotional stress-induced Tako-tsubo cardiomyopathy: animal model and molecular mechanism. Ann N Y Acad Sci. 2004;1018:437-444. 15240400 10.1196/annals.1296.054

[4] Kurowski V Kaiser A von Hof K Apical and midventricular transient left ventricular dysfunction syndrome (Tako-tsubo cardiomyopathy): frequency, mechanisms, and prognosis. Chest. 2007;132:809-816. 17573507 10.1378/chest.07-0608

[5] Kume T Kawamoto T Okura H Local release of catecholamines from the hearts of patients with Tako-tsubo-like left ventricular dysfunction. Circ J. 2008;72:106-108. 18159109 10.1253/circj.72.106

[6] Giavarini A Chedid A Bobrie G Plouin PF Hagege A Amar L Acute catecholamine cardiomyopathy in patients with phaeochromocytoma or functional paraganglioma. Heart (British Cardiac Society). 2013;99:1438-1444. 23837998 10.1136/heartjnl-2013-304073

[7] Wittstein IS Thiemann DR Lima JA Neurohumoral features of myocardial stunning due to sudden emotional stress. N Engl J Med. 2005;352:539-548. 15703419 10.1056/NEJMoa043046

[8] Abraham J Mudd JO Kapur NK Klein K Champion HC Wittstein IS Stress cardiomyopathy after intravenous administration of catecholamines and beta-receptor agonists. J Am Coll Cardiol. 2009;53:1320-1325. 19358948 10.1016/j.jacc.2009.02.020

[9] Mauriello A Rovella V Anemona L Increased sympathetic renal innervation in hemodialysis patients is the anatomical substrate of sympathetic hyperactivity in end-stage renal disease. J Am Heart Assoc. 2015;4:e002426. 26611731 10.1161/JAHA.115.002426 PMC4845297

[10] Augustyniak RA Tuncel M Zhang W Toto RD Victor RG Sympathetic overactivity as a cause of hypertension in chronic renal failure. J Hypertens. 2002;20(1):3-9. 11791019 10.1097/00004872-200201000-00002

[11] Poorgholami F Koshkaki AR Kargar Jahromi M Parniyan R A study of the influence of group-based learning of stress management on psychology symptoms levels of hemodialysis patients. Glob J Health Sci. 2016;8(11):52525.

[12] Mapes DL Bragg-Gresham JL Bommer J Health-related quality of life in the Dialysis Outcomes and Practice Patterns Study (DOPPS). Am J Kidney Dis. 2004;44(5 suppl 2):54-60. 15486875 10.1053/j.ajkd.2004.08.012

[13] González-De-Jesús LN Sánchez-Román S Morales-Buenrostro LE Assessment of emotional distress in chronic kidney disease patients and kidney transplant recipients. Rev Invest Clin. 2011;63:558-563. 23650668

[14] Tsuchihashi K Ueshima K Uchida T Transient left ventricular apical ballooning without coronary artery stenosis: a novel heart syndrome mimicking acute myocardial infarction. J Am Coll Cardiol. 2001;38:11-18. 11451258 10.1016/s0735-1097(01)01316-x

[15] Fukui M Mori Y Tsujimoto S “Takotsubo” cardiomyopathy in a maintenance hemodialysis patient. Ther Apher Dial. 2006;10:94-100. 16556144 10.1111/j.1744-9987.2006.00308.x

[16] Muratsu J Morishima A Ueda H Hiraoka H Sakaguchi K Takotsubo cardiomyopathy in two patients without any cardiac symptom on maintenance hemodialysis. Case Rep Nephrol. 2013;2013:640976. 24527248 10.1155/2013/640976 PMC3914169

[17] Kusaba T Sasaki H Sakurada T Takotsubo cardiomyopathy thought to be induced by MRSA meningitis and cervical epidural abscess in a maintenance-hemodialysis patient: case report [in Japanese]. Nihon Jinzo Gakkai Shi. 2004;46:371-376. 16773801

[18] Takemoto F Chihara N Sawa N Takotsubo cardiomyopathy in a patient undergoing hemodialysis. Kidney Int. 2009;76(4):467. 19644490 10.1038/ki.2009.127

[19] Shin MJ Rhee H Kim IY Clinical features of patients with stress-induced cardiomyopathy associated with renal dysfunction: 7 case series in single center. BMC Nephrol. 2013;14:213. 24099436 10.1186/1471-2369-14-213 PMC3852228

